# The Role of Centrosome Misorientation and miR‐1270 in Impaired Endothelial Cell Migration in Preeclampsia

**DOI:** 10.1096/fj.202502468R

**Published:** 2025-09-16

**Authors:** Bianca Schröder‐Heurich, Nadia Meyer, Katja Richter, Constantin S. von Kaisenberg, Frauke von Versen‐Höynck

**Affiliations:** ^1^ Gynecology Research Unit Hannover Medical School Hannover Germany; ^2^ Department of Obstetrics and Gynecology Hannover Medical School Hannover Germany

**Keywords:** cell migration, centrosome orientation, ECFC, endothelial progenitor cells, miR‐1270, preeclampsia

## Abstract

Preeclampsia is a serious pregnancy‐related disorder that poses significant health risks to the mother and offspring. A key aspect of preeclampsia is impaired endothelial cell migration, which is essential for the repair and integrity of the vasculature. Preeclamptic endothelial progenitor cells (EPC) exhibit defects in migration, although the precise molecular mechanisms underlying these defects remain unclear. Cellular spatial organization, such as centrosome orientation, regulates the migration process by influencing cell polarity and movement. Epigenetic changes in preeclampsia may disrupt these processes and thus impair endothelial function. We recently described the role of microRNA (miR)‐1270 in preeclamptic EPC, linking its dysregulated expression to impaired EPC motility. Here, we demonstrate that endothelial cell migration defects in preeclampsia, along with reduced miR‐1270 levels, are accompanied by changes in centrosome orientation. This study provides novel insights into the molecular consequences of preeclampsia and its detrimental effects on endothelial dynamics.

## Graphical abstract

During wound healing, centrosome orientation toward the wound is crucial for directed cell migration. Epigenetic modifications in preeclampsia can disrupt this orientation, leading to centrosome misorientation. This misorientation affects the cell's ability to migrate efficiently, potentially impairing the healing process. Understanding these molecular mechanisms is vital for developing targeted therapies to enhance wound repair.

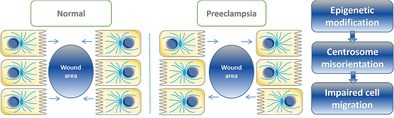

## 1 Introduction

Preeclampsia is a severe disorder of pregnancy, endangering the health of both the mother and the offspring [1]. Characterized by hypertension, preeclampsia can lead to serious complications, including long‐term cardiovascular disease [2]. Despite intensive research, the exact mechanisms that lead to the development of preeclampsia are not fully understood. Epigenetic changes are increasingly recognized for their significant role, as they can exert lasting effects on cell function and tissue structure. One particularly important aspect is endothelial cell migration, an essential process for blood vessel formation and repair, which can be significantly affected by preeclampsia [3, 4].

Endothelial cell migration is regulated by a number of cellular components, including the cytoskeleton and, in particular, the orientation of the centrosomes [5]. By supporting the asymmetric distribution of cellular structures, the centrosome acts as a microtubular organizing center and determines the direction of cell movement [6]. Under normal conditions, the centrosome enables efficient processes, for example, wound healing, by controlling the polarity and directional migration of cells [7]. It can be assumed that the epigenetic changes induced by preeclampsia may lead to a disruption of these regulatory mechanisms, thereby affecting the ability of endothelial cells to migrate.

Epigenetic modifications include regulation of non‐coding RNAs like microRNAs (miR), which influence cell function and thus can contribute to defective cell differentiation and migration processes [8]. This phenomenon is particularly evident in preeclampsia, where endothelial colony forming cells (ECFC)—a subtype of endothelial progenitor cells (EPC)—exhibit impaired migration, previously associated with miR‐1270 downregulation [9, 10].

Misorientation of the centrosome can disrupt cell polarity and reduce cell motility, which could severely impair wound healing and vascularization. In this context, centrosome‐driven cell migration under preeclamptic conditions is becoming increasingly important. This study examines whether preeclampsia‐induced downregulation of miR‐1270 in ECFC is associated with centrosome misorientation and impaired endothelial migration. This knowledge may provide new approaches for therapeutic intervention and a better understanding of the pathophysiological mechanisms of preeclampsia.

## 2 Material and Methods

### 2.1 ECFC Isolation and Cultivation

After written informed consent was obtained from each participant, umbilical cord blood was collected from different donors (control and preeclamptic pregnancies). Approval was acquired from the Institutional Review Board (ethical approval number 3254). Isolation, characterization, and cultivation of ECFC were performed as previously described [10].

### 2.2 MiR‐1270 Transfection

Transfection of control ECFC with miR‐1270 inhibitor and related negative control (Neg Ctrl) was performed as previously reported by us [10].

### 2.3 Migration Assay

A migration assay was carried out using ECFC from preeclamptic pregnancies and after miR‐1270 inhibition, comparing the results to those of control ECFC. Confluence of ECFC was reached by 5 × 10^4^ cells that had been seeded in duplicates in 24‐well culture plates and cultured until monolayer formation. The monolayer was scratched with a sterile P200 pipette tip, washed twice with phosphate‐buffered saline (PBS) and cultured in fresh medium containing 7.5% fetal bovine serum (FBS) and 1% Penicillin/Streptomycin (P/S). Phase contrast images were taken immediately after scratching and after 18 h using a Leica DMI 6000 B microscope (Leica). The scratch areas were quantified using ImageJ 1.50b software (National Institute of Health).

### 2.4 Centrosome Number Quantification and Orientation Assay

To determine the centrosome number per cell population, 1 × 10^5^ cells were seeded on sterile cover glasses in 6‐well plates and grown to 60%–70% confluence. After fixation with 3% formaldehyde/2% sucrose and permeabilization with Triton X‐100 0.2% (Sigma Aldrich) cells were stained with a ɣ‐tubulin antibody (1:500; Sigma Aldrich) and 4′,6‐diamidino‐2‐phenylindole (DAPI) (Thermo Fisher Scientific). For each cell population, 150 cells were randomly counted to determine the number of centrosomes per cell. Giant cells and cells with micronuclei were excluded from counting.

To analyze the centrosome orientation during the migration process, preeclamptic, miR‐1270‐inhibited, and control ECFC were cultured on coverslips in 6‐well culture plates until a stable monolayer was formed. A wound was created 24 h later using a sterile P200 pipette tip. The cells were washed twice with PBS, cultured in fresh medium for 1 h, fixed, and permeabilized as described above. After incubation for 2 h with a pericentrin antibody (1:500; Abcam) in 2% normal goat serum/PBS, counterstaining was performed with a secondary antibody (Alexa Fluor anti‐mouse IgG 546) for 2 h. For counterstaining of the actin cytoskeleton and DNA, Phalloidin FITC (1:500; Thermo Fisher) and DAPI were used. Randomly selected images were taken along the wound area, and the orientation of the centrosomes of the cells was determined manually. Only cells that were directly located at the front line toward the scratch were evaluated for centrosome orientation. Between 51 and 85 cells per biological replicate were counted, and the proportion of cells with forward‐oriented and backward‐oriented centrosomes was determined from the total.

### 2.5 Statistical Analysis

All experiments were performed with at least *n* = 4 biological replicates unless otherwise indicated. After testing the data for normal distribution using the Shapiro–Wilk test, various statistical methods were applied, including Welch's *t*‐test and Mann–Whitney *U* test, each according to the requirements of the analysis.

## 3 Results

**FIGURE 1:**
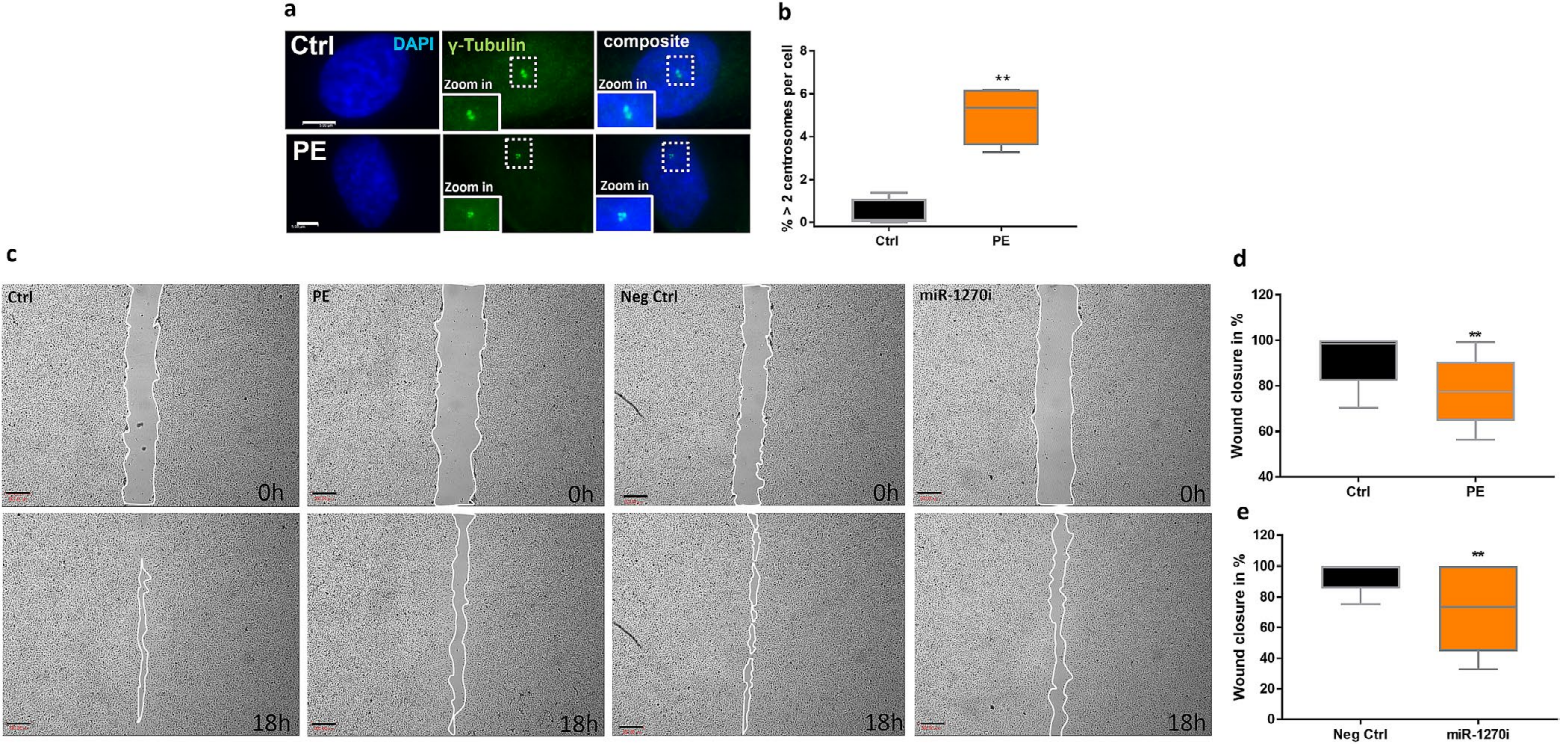
Centrosome number and impaired migration of miR‐1270‐inhibited and preeclamptic ECFC. (a) Representative images of preeclamptic and control ECFC. Centrosomes were counterstained with ɣ‐tubulin (green), DNA with DAPI (blue); Scale = 5 μm. (b) Quantification of centrosome amplification (c). Representative images of a migration assay for preeclamptic, miR‐1270‐inhibited and control ECFC. (d, e) Wound closure of preeclamptic (PE) and miR‐1270‐inhibited (miR‐1270i) ECFC. Scale = 500 μm; (*n* = 5 control ECFC; *n* = 4 preeclamptic ECFC). Ctrl, Control; Neg Ctrl, Negative control. **p < 0.01.

As previously reported, ECFC isolated from preeclamptic pregnancies demonstrate a significantly reduced migration potential [9]. Effective migration requires the coordinated movement of cells toward a target location. However, an increased number of centrosomes has been shown to negatively affect migration, primarily through their effects on cell polarity and movement [11]. Analyzing the centrosome number in preeclamptic ECFC revealed a significant centrosome amplification, with a fold‐change increase of 10.8 compared to the control group (*p* = 0.003) (Figure 1a,b).

**FIGURE 2:**
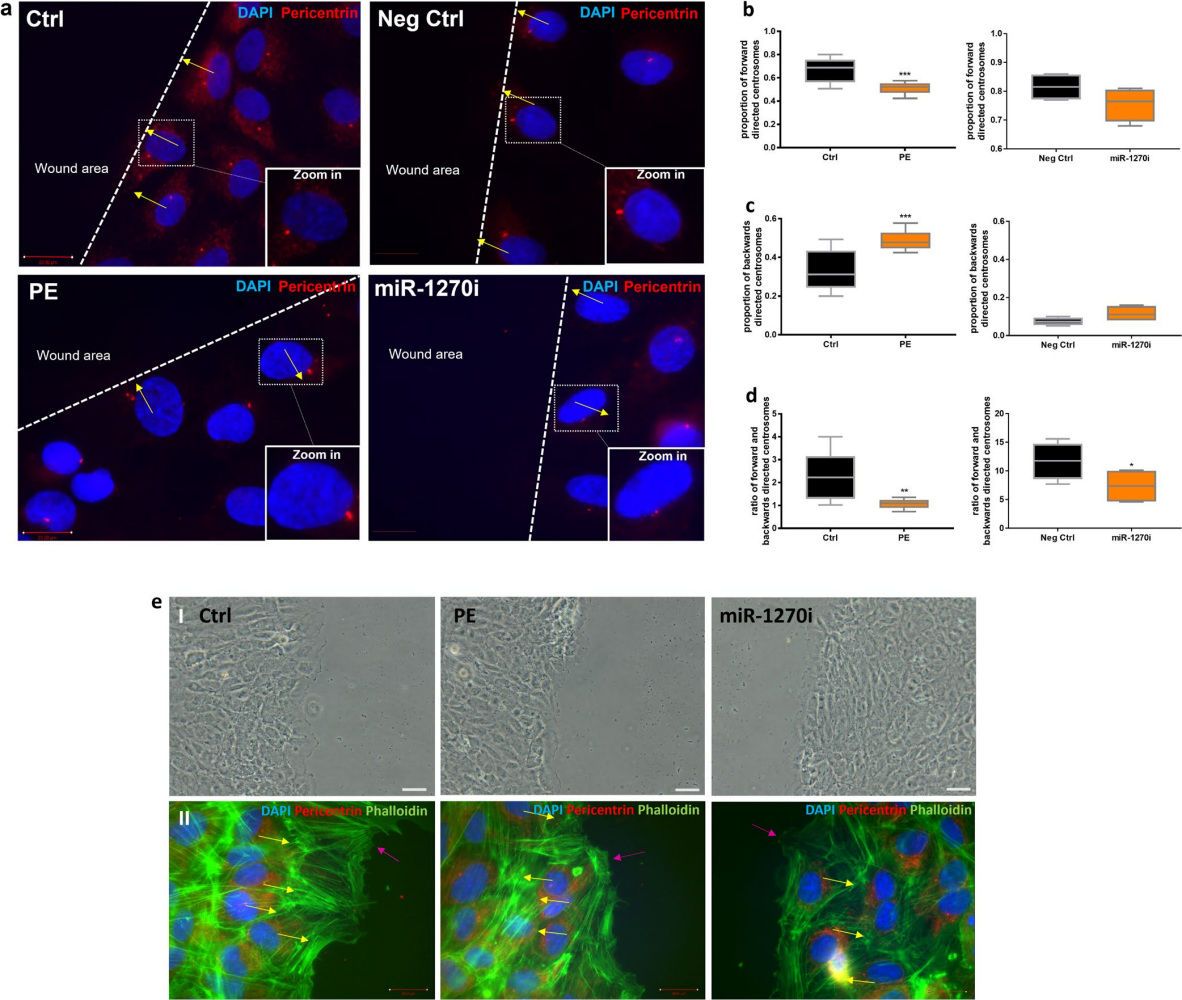
Centrosome orientation of miR‐1270‐inhibited and preeclamptic ECFC in a migration assay. (a) Representative images of forward‐ and backward‐oriented centrosomes (stained with pericentrin (red)) of preeclamptic (PE) and miR‐1270‐inhibited (miR‐1270i) ECFC and related controls. DNA was counterstained with DAPI (blue). (b) Quantification of forward, (c) backward and (d) ratio of forward–backward‐directed centrosomes toward the wound area of preeclamptic and miR‐1270‐inhibited ECFC. (e) I: Representative phase contrast images of control, preeclamptic (PE) and miR‐1270‐inhibited (miR‐1270i) ECFC (*n* = 2 technical replicates). Scale = 100 μm. II: Representative images of actin cytoskeleton (green) organization and filopodia extension area (*n* = 2 technical replicates). Yellow arrow = direction of centrosome orientation. Purple arrow = filopodia area next to the wound area. Scale = 20 μm; *n* = 4 control ECFC; *n* = 4 preeclamptic ECFC. Ctrl, Control; Neg Ctrl, Negative control. *p < 0.05, **p < 0.01. ***p < 0.005.

Our recent study demonstrated that dysregulation of miR‐1270 contributes to impaired cell motility in the context of preeclampsia [12]. To further investigate the migratory processes influenced by miR‐1270 in preeclampsia, we compared the migration ability of preeclamptic‐ and miR‐1270‐inhibited ECFC. Our results showed that both miR‐1270‐inhibited and preeclamptic ECFC exhibited similar impaired wound closure compared to control ECFC (Figure 1c) (relative re‐migrated area: miR‐1270 ECFC: 70.87 ± 5.11 vs. control ECFC: 93.43 ± 2.03, *p* = 0.006; preeclamptic ECFC: 78.49 ± 3.78 vs. control ECFC: 92.04 ± 2.51, *p* = 0.006) (Figure 1d,e). Pericentrin staining was employed to evaluate centrosome orientation during the migration process, allowing for assessment of pro‐migratory centrosome alignment in both groups (Figure 2a). Preeclamptic ECFC displayed reduced proportions of forward‐oriented centrosomes toward the wound (preeclamptic ECFC: 0.51 ± 0.014 vs. control: 0.66 ± 0.03, *p* = 0.005) and increased proportions of backward‐oriented centrosomes (preeclamptic ECFC: 0.48 ± 0.02 vs. control ECFC: 0.3 ± 0.03, *p* = 0.005). MiR‐1270‐inhibited ECFC showed decreased proportions of forward (miR‐1270‐inhibited ECFC: 0.76 ± 0.03 vs. control: 0.82 ± 0.02, *p* = 0.08) and backward‐oriented centrosomes (miR‐1270‐inhibited ECFC: 0.11 ± 0.02, vs. control ECFC: 0.07 ± 0.01, *p* = 0.07), but this effect was not significant (Figure 2b,c). The ratio of forward‐to‐backward‐directed cells was reduced in preeclamptic (1.07 ± 0.06) versus control (2.29 ± 0.34, *p* = 0.003) and miR‐1270‐inhibited ECFC (7.36 ± 1.51 vs. control: 11.72 ± 1.61, *p* = 0.05), which may contribute to the undirected endothelial cell migration (Figure 2d). To assess potential morphological differences between control, preeclamptic, and miR‐1270‐inhibited ECFC in a wound healing assay, phase contrast imaging was performed of the wound area (Figure 2e,I). These images revealed no visible significant differences in overall cell morphology between the groups. However, staining of the actin cytoskeleton uncovered distinct differences in cytoskeletal dynamics: control ECFC exhibited larger areas where filopodia extended further into the wound area compared to preeclamptic and miR‐1270‐inhibited ECFC. Notably, these differences were not uniformly distributed across all regions of the wound; a representative example is shown in Figure 2e,II.

## 4 Discussion

This study investigates the impact of preeclampsia and the subsequent downregulation of miR‐1270 on the migration of ECFC, highlighting the critical role of centrosome orientation. Here we demonstrate that preeclamptic ECFC exhibit both aberrant centrosome number and misoriented positioning, characterized by a reduction in forward‐oriented and an increase in backward‐oriented centrosomes. MiR‐1270 has been reported to exhibit both pro‐ and anti‐migratory effects depending on the cellular context [12, 13]. In our study, inhibition of miR‐1270 in control ECFC exhibits similar defects in migration and centrosome orientation as preeclamptic ECFC. These findings suggest that miR‐1270 downregulation in preeclampsia contributes to impaired ECFC migration through disruption of centrosome polarity. The results of this study highlight the importance of epigenetic changes in preeclampsia, particularly the role of miR‐1270 in modulating cytoskeletal organization and endothelial cell dynamics. Specifically, miR‐1270 may be pivotal in disrupting vascular homeostasis in preeclampsia, contributing to significant implications for the function of the affected tissues. This provides novel insights into the molecular consequences of preeclampsia and its impact on endothelial homeostasis.

## Author Contributions

B. Schröder‐Heurich: conceived and designed the research, manuscript writing. B. Schröder‐Heurich, Nadia Meyer, Katja Richter: performed the research, acquired and analyzed the data. Constantin S. von Kaisenberg: provision of study material or patients, final approval of manuscript. Frauke von Versen‐Höynck: financial support, administrative support, manuscript writing, and final approval of manuscript. All authors were involved in drafting and revising the manuscript.

## Ethics Statement

This study was conducted in accordance with the principles of the Declaration of Helsinki and was approved by the Ethics Committee of Hannover Medical School (Ethical Approval Number 3254).

## Conflicts of Interest

The authors declare no conflicts of interest.

## Data Availability

The data that support the findings of this study are available on request from the corresponding author.
